# Within- and between-day test–retest reliability of responses to rapid bilateral anterolateral magnetic phrenic nerve stimulation in healthy humans (ReStim)

**DOI:** 10.3389/fphys.2025.1481766

**Published:** 2025-02-11

**Authors:** Kyle G. P. J. M. Boyle, Andrea A. Beglinger, Heinrich Häusler, Anna Stahel, Esther I. Schwarz, Christina M. Spengler

**Affiliations:** ^1^ Exercise Physiology Lab, Institute of Human Movement Sciences and Sport, ETH Zurich, Zurich, Switzerland; ^2^ Faculty of Medicine, University of Zurich, Zurich, Switzerland; ^3^ Department of Pulmonology, University Hospital Zurich, University of Zurich, Zurich, Switzerland; ^4^ Zurich Center for Integrative Human Physiology (ZIHP), University of Zurich, Zurich, Switzerland

**Keywords:** rapid magnetic stimulation, phrenic nerve stimulation, reliability, diaphragm contraction, tidal volume, transdiaphragmatic pressure, non-invasive ventilation, diaphragm atrophy

## Abstract

**Background:**

Mechanical ventilation can lead to lung injury and diaphragmatic dysfunction. Rapid bilateral anterolateral magnetic phrenic nerve stimulation (rBAMPS) may attenuate both of the aforementioned issues by inducing diaphragm activation. However, in order for rBAMPS to become part of standard of care, the reliability of inspiratory responses to rBAMPS needs to be established.

**Methods:**

Eighteen healthy participants (9F) underwent five blocks of 1-s rBAMPS at 25 Hz starting at 20% of maximal stimulator output with 10% increments. Three blocks were completed on the same day to test within-day reliability, and two additional blocks were each completed on subsequent days to test between-day reliability. Mean transdiaphragmatic pressure (P_di,mean_), tidal volume (V_T_), discomfort, pain, and paresthesia were recorded for each rBAMPS. Relative and absolute reliability of both P_di,mean_ and V_T_ were quantified by calculating intraclass correlation coefficients (ICC) and standard error of measurements (SE_M_), respectively. An ordinal regression was used to determine changes of sensory ratings within and between days.

**Results:**

At all stimulator outputs, within-day P_di,mean_ displayed “good” reliability (ICC range 0.78–0.89). Between days, P_di,mean_ reliability was also “good” (ICC range 0.79–0.87) at stimulator outputs of 20%–50% of maximum, but “moderate” (ICC range 0.56–0.72) at stimulator outputs of 60%–100%. SE_M_ for P_di,mean_ within day ranged from 0.9 to 3.4 across tested stimulator outputs and increased on average by 1.4 ± 0.9 between days. The V_T_ reliability was “good” to “excellent” within (ICC range 0.82–0.94) and between (ICC range 0.81–0.96) days at all stimulator outputs. SE_M_ for V_T_ within day ranged from 0.08 to 0.36 and from 0.11 to 0.30 between days and tended to be larger at stimulator outputs greater than 50% of maximum. Subsequent blocks within day were associated with decreased discomfort and pain (P ≤ 0.043), while subsequent days were associated with decreased discomfort and paresthesia (P < 0.001).

**Discussion:**

rBAMPS appears to induce reliable diaphragmatic contractions, while select sensory responses become blunted over repeated stimulations. However, as reliability is slightly lower between days compared to within day, stimulation parameters may need to be adjusted to achieve similar responses on different days.

## 1 Introduction

Despite the necessity to treat respiratory failure, mechanical ventilation, particularity positive pressure ventilation, is not without its drawbacks. Although not an exclusive list, two possible glaring consequences of mechanical ventilation include the following: 1) ventilator-induced lung injury (VILI) caused by positive pressure that generates stress and strain on the lungs (Slutsky and Ranieri, 2014) and 2) ventilator-induced diaphragmatic dysfunction (VIDD) caused by the inactivity of the respiratory muscles (Vassilakopoulos and Petrof, 2004). Diaphragmatic atrophy is associated with a reduced ability for volitional inspiration and thus is associated with the patient’s prolonged reliance on mechanical ventilation (Dres et al., 2017). Given that diaphragmatic atrophy (a hallmark symptom of VIDD) occurs in as little as 18 h (Levine et al., 2008), the implementation of an early, safe, and reliable intervention is paramount for a positive patient prognosis.

Inducing diaphragm contractions *via* neurostimulation during mechanical ventilation may attenuate both aforementioned drawbacks by reducing the diaphragm’s inactivity, while simultaneously reducing the pressure required by mechanical ventilation by generating negative intrathoracic pressure swings that are more representative of physiological breathing. In fact, neurostimulation during mechanical ventilation has been shown to protect against diaphragm atrophy and dysfunction in animal models (Masmoudi et al., 2013; Yang et al., 2013; Reynolds et al., 2017). In humans, development of new as well as advancements in older technologies have produced a variety of methods to perform diaphragm neurostimulation, as described elsewhere (Etienne et al., 2023). One such promising method, due to its non-invasive nature, is magnetic phrenic nerve stimulation. Although phrenic nerve stimulation cannot replace mechanical ventilation, its addition to mechanical ventilation may provide protection against VILI and VIDD.

Previous research has explored the use of rapid cervical magnetic stimulation (rCMS) (Adler et al., 2011), rapid bilateral anterolateral magnetic phrenic nerve stimulation (rBAMPS) (Sander et al., 2010; Boyle et al., 2022), and rapid anterior magnetic stimulation (raMS) (Boyle et al., 2022) with respect to diaphragm pacing in healthy humans. For instance, recent work by our group showed that raMS could not produce sufficient diaphragm contractions due to the likely co-activation of the brachial plexus, which resulted in excessive chest and shoulder movement, which ultimately shifted the magnetic coil out of position (Boyle et al., 2022). Adler et al. (2011), however, showed that rCMS was capable of producing sufficient diaphragmatic contractions, but with a lack of ventilatory response attributed to upper airway collapse (UAC). On the contrary, rBAMPS has been shown to mitigate (but not alleviate) the occurrence of UAC and produce diaphragm contractions capable of inducing ventilation (Sander et al., 2010; Boyle et al., 2022). Therefore, rBAMPS may serve as the optimal technique to perform non-invasive diaphragm stimulation in an ICU setting.

In order for non-invasive rapid magnetic stimulation to become part of standard of care within the ICU, the reliability of responses needs to be assessed. Although the reliability of various characteristics of diaphragmatic twitch responses has been explored in depth (Bellemare and Bigland-Ritchie, 1984; Mills et al., 1995; Criner et al., 1999; Luo et al., 2002; Welch et al., 2017; Ramsook et al., 2021), the reliability of responses to rapid trains of magnetic stimulation is less clear. Therefore, the present study sought to determine the within- and between-day test–retest reliability of inspiratory responses to rBAMPS in healthy humans (the ReStim study). In addition, the present study explored whether there is a change to various side-effects in response to rBAMPS within, as well as between days.

## 2 Materials and methods

### 2.1 Ethical approval

This study was approved by the Cantonal Ethics Committee of Zurich (Project ID 2020-03033), conformed with the guidelines of the Declaration of Helsinki, and was registered on clinicaltrials.gov (NCT05302752).

### 2.2 Experimental design

**FIGURE 1:**
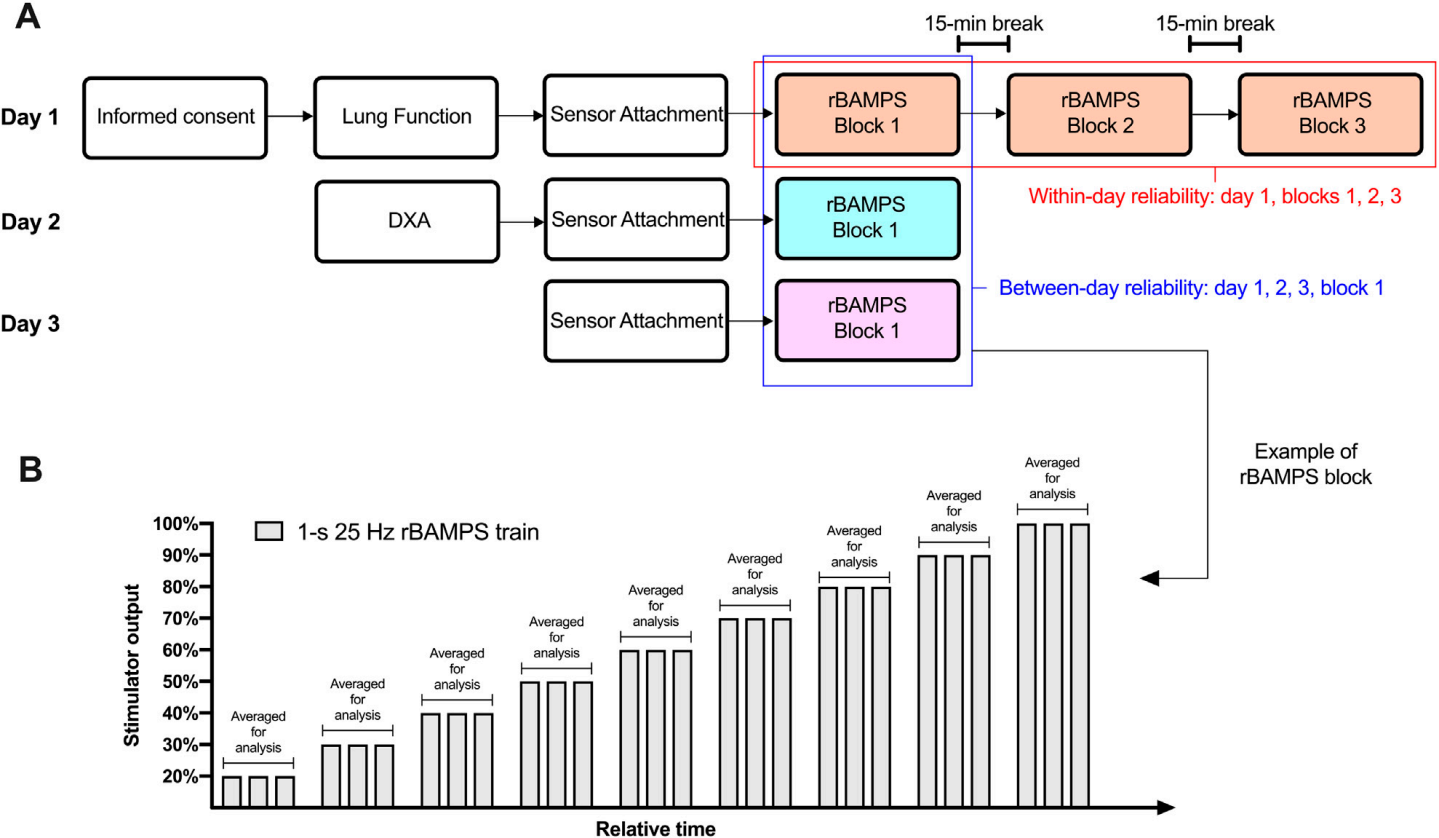
Study protocol. **(A)** Overview of the three study days including all completed tasks and what rapid bilateral anterolateral magnetic phrenic nerve stimulation (rBAMPS) blocks were compared to each other. **(B)** Example of an rBAMPS block that was conducted on all study days. DXA, dual-energy X-ray absorptiometry.

The study took place over three visits to the Exercise Physiology Lab at ETH in Zurich, Switzerland, which can be visualized in Figure 1. Briefly, on the first visit, the participants’ lung function and respiratory muscle strength were evaluated before undergoing three blocks (blocks 1–3) of 1-s rBAMPS at 25 Hz to test within-day test–retest reliability. Each block consisted of at least three trains of rBAMPS at each stimulator output starting at 20% of maximal stimulator output, with increases in 10% increments until 100% was reached, or participant cessation. To test between-day test–retest reliability, participants underwent a single matching block of rBAMPS on day 2 and day 3, which was subsequently compared to block 1 on day 1. During all five stimulation blocks, cardiorespiratory measurements and side-effects were measured throughout, and each visit began with 6 min of resting breathing to quantify baseline measurements. All study visits occurred at the same time of day (within 2 h) at least 24 h apart. Participants were instructed to abstain from caffeine consumption and physical activity on the day of each study visit, as well as intense exercise 48 h before.

### 2.3 Participants

Eighteen healthy individuals participated in the study (9M:9F). Two participants (2F) did not complete visits on day 2 and day 3. Therefore, data for between-day reliability are presented for 16 participants only (9M:7F). Participant characteristics can be found in Table 1.

**TABLE 1:** Participant characteristics.

Anthropometrics		
Sex	9M:9F	
Age, years	25 ± 6	
Height, cm	173.2 ± 8.8	
Body weight, kg	70.1 ± 13.5	
Body fat, %	24 ± 8	
BMI, kg ⋅ m^-2^	23.2 ± 3.0	
Pulmonary function		
FVC, L (% predicted)	5.3 ± 1.1	(118 ± 12)
FEV_1_, L (% predicted)	4.3 ± 0.9	(111 ± 15)
FEV_1_/FVC, % (% predicted)	81 ± 8	(95 ± 9)
PEF, L ⋅ s^-1^ (% predicted)	8.8 ± 2.4	(103 ± 19)
FEF_25-75_, L ⋅ s^-1^ (% predicted)	4.1 ± 1.5	(94 ± 32)
TLC, L (% predicted)	6.4 ± 1.5	(103 ± 13)
FRC, L (% predicted)	3.3 ± 0.8	(110 ± 16)
RV, L (% predicted)	1.1 ± 0.5	(83 ± 29)
Maximal voluntary ventilation, L ⋅ min^-1^ (% predicted)	131.7 ± 33.1	(114 ± 20)
Maximal volitional pressure generation		
Maximal inspiratory mouth pressure, cmH_2_O (% predicted)	99.8 ± 26.8	(108 ± 33)
Maximal expiratory mouth pressure, cmH_2_O (% predicted)	136.7 ± 36.4	(114 ± 34)

BMI, body mass index; FVC, forced vital capacity; FEV_1_, forced expiratory volume in one second; PEF, peak expiratory flow; FEF_25-75_, forced expiratory flow between 25% and 75% of FVC; TLC, total lung capacity; FRC, functional residual capacity; RV, residual volume. Predicted FVC and FEV_1_ values were obtained from Bowerman et al. (2023). Predicted PEF values were obtained from Quanjer et al. (1993). Predicted FEF_25-75_ values were obtained from Quanjer et al. (2012). Predicted TLC, FRC, and RV values were obtained from Hall et al. (2021). Predicted maximal voluntary ventilation was obtained by multiplying FEV_1_ by 35. Predicted maximal inspiratory and expiratory values were obtained from Wilson et al. (1984). Values are mean ± standard deviation.

### 2.4 Lung function and respiratory muscle strength

Standard spirometry and body plethysmography were conducted with a commercially available testing system and body box (Quark PFT and Q-Box, COSMED, Rome, Italy) according to current guidelines (Miller et al., 2005; Wanger et al., 2005), with the participants seated in an upright position. Maximal inspiratory and expiratory pressures (MIP and MEP, respectively) were evaluated according to current guidelines (American Thoracic Society/European Respiratory, 2002; Laveneziana et al., 2019) from residual volume and total lung capacity, respectively, using a respiratory pressure meter (RP Check, MD Diagnostics Ltd., Kent, United Kingdom). Participant values were converted to percentage of predicted for all of MIP and MEP (Wilson et al., 1984), lung volumes (Hall et al., 2021), and spirometry values (Quanjer et al., 1993; Quanjer et al., 2012; Bowerman et al., 2023) using the newly established race-neutral equations for spirometry when possible (Bowerman et al., 2023).

### 2.5 Respiratory responses to rBAMPS

Transdiaphragmatic pressure (P_di_) was assessed using two balloon-tipped catheters (Adult Esophageal Balloon Catheters 47-9005, Cooper Surgical, Trumbull, CT, United States) connected to calibrated differential pressure transducers (DP45, Validyne Engineering, Northbridge, CA, United States). First, the participant’s nasal cavity and throat were numbed with local anesthetic (Xylocaine Spray 10%, Aspen Pharma Schweiz GmbH, Baar, Switzerland) prior to insertion of both balloons one at a time through the same nare into the stomach. Participants were then instructed to perform a Valsalva maneuver to remove the air from the balloons, which were subsequently filled with 1 and 2 mL of air. The balloon with 2 mL of air remained in the stomach to record the gastric pressure (P_ga_), while the balloon with 1 mL of air was placed in the lower third of the esophagus to record the esophageal pressure (P_es_). Specifically, the balloon was withdrawn in 1-cm increments while the participants performed sniff maneuvers until the first instance of a negative deflection occurred. The balloon was then withdrawn 10 cm to ensure its complete removal from the stomach. Participants were then moved into the semi-recumbent position, and the positioning of the esophageal balloon was confirmed with the occlusion test (Baydur et al., 1982), while the gastric balloon was confirmed to be in the stomach by pressing on the abdomen and assessing for a positive pressure deflection. Adjustments in the final positioning of each balloon were made as needed. Both balloons were adhered with a tape to ensure the same position throughout testing, and each balloon was placed in the same position on subsequent days. P_di_ was calculated as P_ga_ - P_es_. Participants were also instrumented with a pneumotachometer to continuously record the flow (Series 3813, Hans Rudolph, Shawnee, KS, United States). Two respiratory belt transducers (TN1132/ST, ADInstruments, Dunedin, New Zealand) that generate a linear voltage (0–100 mV) proportional to changes in length were used to assess changes in thoracic (ΔTC) and abdominal circumference (ΔAC). The thoracic belt was placed over the nipple line in men and directly below the breasts in women. The abdominal belt was placed over the navel in all participants.

### 2.6 Side-effects to rBAMPS

Participants were asked to rate their sensation of discomfort, pain, and paresthesia in response to each train of rBAMPS using a visual scale. The scale ranged from 0 to 10 points, and 0 was anchored as “none,” while 10 was anchored as “maximal” (the maximum the participant could imagine). Changes in the galvanic skin response (ΔGSR) were assessed following each rBAMPS train using a commercially available system (FE116 GSP Amp, ADInstruments, Dunedin, New Zealand). The electrodes used (MLT118F GSR Electrodes, ADInstruments, Dunedin, New Zealand) were placed on the participant’s index and ring fingers on their middle phalanx.

### 2.7 Protocol of rBAMPS blocks

All magnetic stimulations were conducted with a dual-head 46-mm butterfly shaped coil (Cool Twin B-46, MagVenture, Farum, Denmark [max dB/dt = 22 kT/s, 317μs]) connected to a single commercially available magnetic stimulator (MagPro ×100, MagVenture, Farum, Denmark) and an active cooling unit (cooler coil unit + high performance option, MagVenture, Farum, Denmark). All rBAMPS occurred at the end of a passive expiration, and participants were instructed to keep their glottis open, if possible. Stimulation took place with participants lying in a hospital bed in a semi-recumbent position such that their torso was raised to 30°. Participants’ heads were placed in a vacuum cushion (Vacuform^®^ 2.0 vacuum pillow 30 × 40 cm, Synmedic AG, Zurich, Switzerland), with their necks slightly extended. The pillow formed to their head such that head and neck position could be replicated on following visits. Additionally, the positions of the participant’s clavicular notch, anterior superior iliac spine, and patella were recorded in reference to a measuring tape fastened to the side of the hospital bed so that the body position could be replicated.

The coil heads were positioned bilaterally on both anterolateral sides of the neck as described previously (Boyle et al., 2022). Briefly, the initial position for each coil head was the location that yielded the highest P_di_ twitch (P_di,tw_) in response to a single stimulation at 100% of stimulator output. Following this, a series of rBAMPS trains were conducted at 25 Hz and 20%–50% of maximal stimulator output to evaluate co-activation that resulted in excessive movement of the participant’s head, shoulders, and arms. If the responses resulted in excessive movement, as indicated by both the experimenters and participant (such that the participant could not tolerate higher stimulator outputs), the location that resulted in the next highest P_di,tw_ was tested in a similar matter. Once the optimal stimulation location was determined, the coils were secured with custom-made lever arms. The location of the coil heads was marked with a skin marker on the participant’s neck, photographed, and measured in reference to their clavicular notch and angle of their lower mandible in order to replicate the position between successive stimuli and days.

Each rBAMPS block consisted of at least three rBAMPS trains at each stimulator output starting at 20% of the maximal intensity of the stimulator with increases in 10% increments. All rBAMPS were 1 s in duration at a stimulation frequency of 25 Hz and occurred at least 30 s apart. Three rBAMPS blocks (blocks 1, 2, and 3) were conducted on visit 1 to test within-day reliability, with 15 min between each. A single identical block of rBAMPS was conducted on both day 2 and day 3 to test the between-day reliability. All rBAMPS trains and P_di,tw_ assessments were conducted at functional residual capacity, which was assessed by comparing end-expiratory P_es_ prior to the stimulation with that of baseline resting breathing values.

### 2.8 Data acquisition and analysis

Physiological measurements were converted from analog to digital with two 16-channel data acquisition systems (PowerLab 16/35, ADInstruments, Dunedin, New Zealand) and collected using LabChart software (Version 8, ADInstruments, Dunedin, New Zealand) with a sampling frequency of 2,000 Hz. The integral of the inspiratory flow was taken to calculate the tidal volume (V_T_), which was then body temperature pressure saturated corrected. Data were analyzed using a custom-written LabChart macro that calculated the mean or peak changes in variables within specific analysis windows. The analysis window in rBAMPS that resulted in a V_T_ response spanned from the onset of stimulation until the absence of inspiratory flow. During rBAMPS that resulted in no activity or UAC, the analysis window was 1 s following the onset of stimulation. ΔGSR was taken within a 10-s analysis window from the onset of stimulation regardless of the response type. Relative mean and peak changes in P_di_ (P_di,mean_ and P_di,peak_); relative peak ΔTC, ΔAC, and ΔGSR; and absolute V_T_ responses were used for statistical analysis. Finally, the responses to individual rBAMPS trains at matching stimulator outputs within a block were averaged together to compare to subsequent blocks (Figure 1B). Within each participant, only stimulator outputs in which the participant tolerated all three rBAMPS trains across all within-day or between-day blocks were used for analysis.

### 2.9 Statistics

Test–retest reliability of rBAMPS was assessed by calculating intraclass correlation coefficients (ICC) for P_di,mean_, P_di,peak_, V_T_, ΔTC, and ΔAC within (blocks 1, 2, and 3 on day 1) and between days (block 1 of days 1, 2, and 3) at each stimulator output using a two-way mixed-effects model testing for absolute agreement. ICC values were interpreted as poor (ICC <0.5), moderate (ICC = 0.50–0.75), good (ICC = 0.75–0.90), and excellent (ICC >0.90) reliability according to Koo and Li (2016). Standard error of measurements (SE_M_) at each stimulator output was calculated in original units as standard deviation • $\sqrt{1-\text{ICC}}$ to provide an absolute index of reliability. The minimal detectable change (MDC) was calculated in original units as 1.96 • SE_M_ • $\sqrt{2}$ . Within-participant coefficients of variation (CV) were calculated between stimuli within blocks. Differences in P_di,mean_, V_T_, ΔTC, ΔAC, and ΔGSR within and between days were tested using a two-way (stimulator output [20%–100%] x within- or between-day rBAMPS blocks [within day: block 1, 2, and 3 on day 1; between days: block 1 on day 1, 2, and 3]) mixed-effects model with repeated measures. Tukey’s *post hoc* tests were conducted in the event of significant main effects when applicable. Discomfort, pain, and paresthesia were analyzed in two ways. First, sensory ratings were treated as ordinal data (from 0 to 10), and an ordinal regression was used to test for associations between sensory ratings with stimulator output, as well as subsequent blocks within and between days. Given the data for each sensory rating was zero inflated, a binary regression was also performed to determine the associations between stimulator output and subsequent blocks within and between days with participants selecting a value greater than 3 points for each sensory rating. A threshold value of 3 was chosen based on previous work attempting to determine the optimal stimulation frequency and stimulator output to perform rapid magnetic stimulation by Boyle et al. (2022) and Adler et al. (2011). Finally, a logistic regression was used to determine the odds of experiencing an UAC with increasing stimulator outputs, as well as within- and between-day rBAMPS blocks. Statistical significance was set at P < 0.05, and all analyses were conducted with Jamovi (v2.3, Sydney, Australia) or Prism (v8.3.1, GraphPad Software, San Diego, CA, United States). Group and grand mean values are expressed as mean ± standard deviation unless stated otherwise.

## 3 Results

### 3.1 Reliability at matched stimulator outputs

**FIGURE 2:**
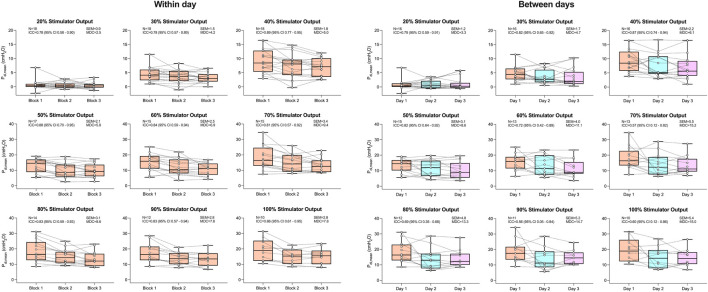
Mean transdiaphragmatic pressure generation (P_di,mean_) in response to rapid bilateral anterolateral magnetic phrenic nerve stimulation. Single-participant data points are represented by white circles connected by dotted lines. Upper and lower bands of the underlying box plots represent the interquartile ranges, while the black line represents the median. Box plot whiskers represent min and max values. Number of participants, intraclass correlation coefficients with 95% confidence intervals, standard error of measurement, and minimal detectable change are presented in text at each stimulator output.

P_di,mean_ in response to rBAMPS at all tested stimulator outputs along with associated ICC (95% confidence intervals), SE_M_, and MDC values, within day and between day is presented in Figure 2. The within-day grand means for P_di,mean_ were 11.4 ± 4.8, 9.1 ± 3.8, and 8.4 ± 3.4 cmH_2_O during blocks 1, 2, and 3, respectively. There was a significant effect of stimulator output (P < 0.0001) and block on within-day P_di,mean_ (P < 0.001). *Post hoc* testing revealed that P_di,mean_ within day was significantly higher on block 1 when compared to block 2 from 40% to 100% of maximal stimulator output (all P < 0.038), and when compared to block 3, from 40% to 80% of maximal stimulator output (all P < 0.023). No significant differences were detected between within-day blocks 2 and 3 at any tested stimulator output. Within-day P_di,mean_ displayed “good” reliability (ICC range 0.78–0.89) at all tested stimulator outputs. Between-day, P_di,mean_ grand means were 11.5 ± 4.9 cmH_2_O on day 1, 9.1 ± 4.9 cmH_2_O on day 2, and 8.8 ± 4.5 cmH_2_O on day 3. A significant effect of stimulator output (P < 0.0001) and day (P = 0.020) was detected on between-day P_di,mean_. However, *post hoc* tests revealed only a significant difference between days 1 and 3 at 40% of stimulator output (P = 0.03), but no difference on any other day and stimulator output combination (all P > 0.083). Between-day P_di,mean_ displayed “good” reliability from 20% to 50% of maximal stimulator output (ICC range 0.79–0.87), but “moderate” reliability at stimulator outputs from 60% to 100% (ICC range 0.56–0.72). SE_M_ ranged from 0.9 to 3.4 within day and increased on average by 1.4 ± 0.9 between days. The MDC ranged from 2.5 to 9.4 within day and 3.3 to 15.2 between days. The within-participant within-block CV was 82% at 20% of maximal stimulator output, 42% at 30% of maximal stimulator output, and ranged from 7% to 17% at 40% to 100% of maximal stimulator output. The reliability of P_di,peak_ was similar to that of P_di,mean_ and can be found in the supplemental material of this paper.

**FIGURE 3:**
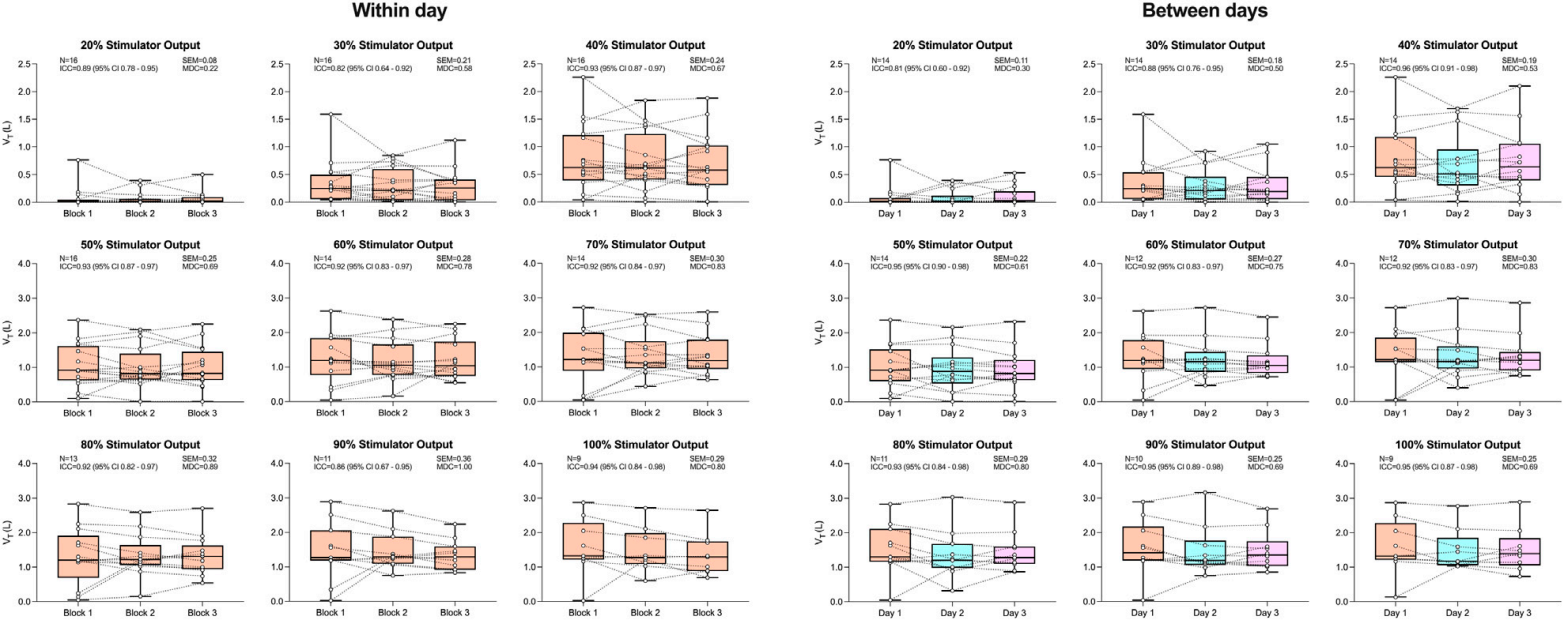
Tidal volume in response to rapid bilateral anterolateral magnetic phrenic nerve stimulation. Single-participant data points are represented by white circles connected by dotted lines. Upper and lower bands of the underlying box plots represent the interquartile ranges, while the black line represents the median. Box plot whiskers represent min and max values. Number of participants, intraclass correlation coefficients with 95% confidence intervals, standard error of measurement, and minimal detectable change are presented in text at each stimulator output.

Figure 3 displays V_T_ in response to rBAMPS reliability both within and between days. Within-day V_T_ grand mean responses were 0.88 ± 0.62 (block 1), 0.87 ± 0.51 (block 2), and 0.85 ± 0.49 L (block 3), while between-day grand means were 0.90 ± 0.63 (day 1), 0.83 ± 0.59 (day 2), and 0.86 ± 0.55 L (day 3). A significant effect of stimulator output on V_T_ was detected both within and between days (both P < 0.0001). No significant effects of within- (P = 0.680) or between-day (P = 0.438) rBAMPS blocks were detected on V_T_. ICCs for V_T_ were good to excellent at all stimulator outputs, both within (range 0.82–0.94) and between days (range 0.81–0.96). Within-day V_T_ SE_M_ values ranged 0.08–0.25 from 20% to 50% of maximal stimulator output and 0.28–0.36 at stimulator outputs from 60% to 100%. V_T_ SE_M_ values between days from 20% to 50% of maximal stimulator output were 0.11–0.22, and were 0.25–0.30 from 60% to 100% of maximal stimulator output. The MDC values were 0.22–1.0 within day and 0.30–0.83 between days. Within-block CVs were 55%, 51% and 31% at 20%, 30%, and 40% of maximal stimulator output, respectively, and were 10%–19% from 50% to 100% of maximal stimulator output.

**FIGURE 4:**
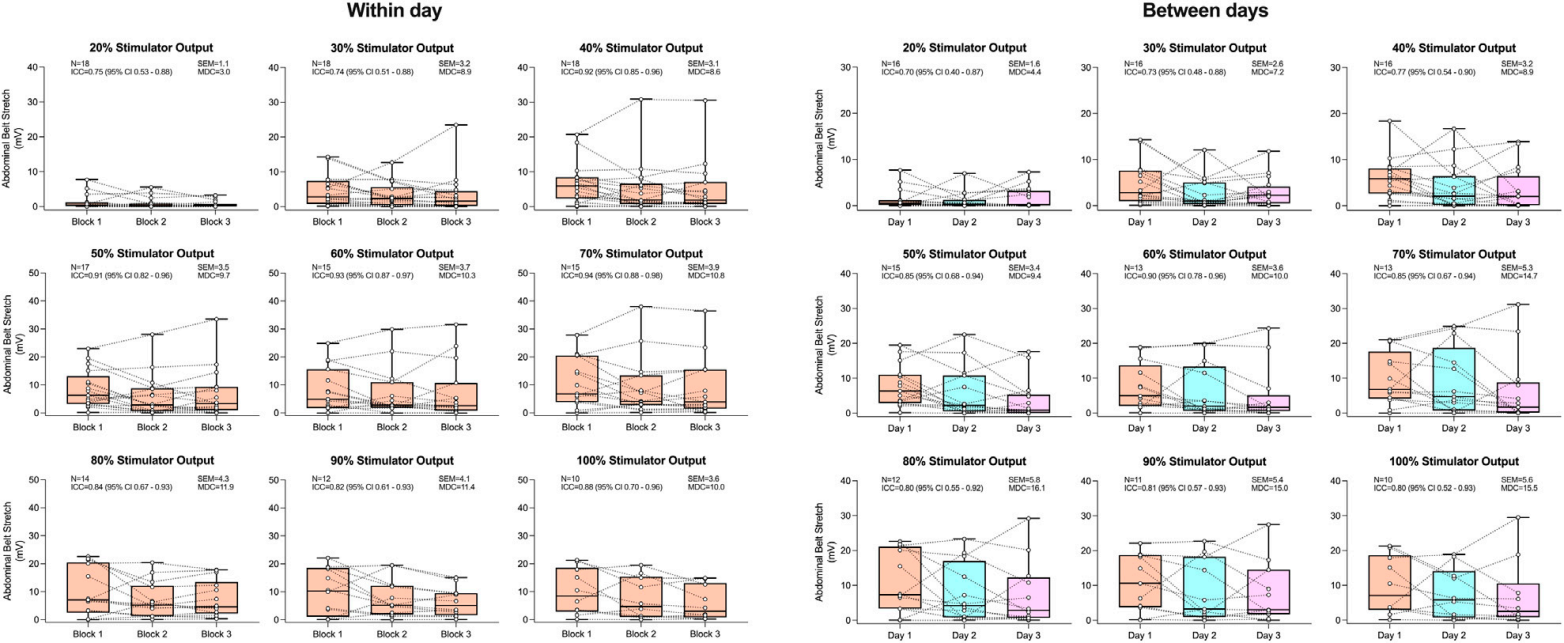
Abdominal belt stretch in response to rapid bilateral anterolateral magnetic phrenic nerve stimulation. Single-participant data points are represented by white circles connected by dotted lines. Upper and lower bands of the underlying box plots represent the interquartile ranges, while the black line represents the median. Box plot whiskers represent min and max values. Number of participants, intraclass correlation coefficients with 95% confidence intervals, standard error of measurement, and minimal detectable change are presented in text at each stimulator output.

**FIGURE 5:**
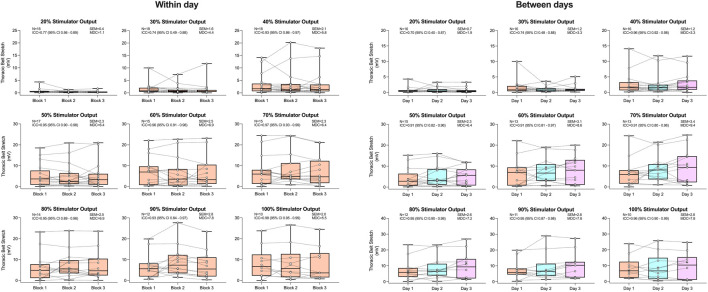
Thoracic belt stretch in response to rapid bilateral anterolateral magnetic phrenic nerve stimulation. Single-participant data points are represented by white circles connected by dotted lines. Upper and lower bands of the underlying box plots represent the interquartile ranges, while the black line represents the median. Box plot whiskers represent min and max values. Number of participants, intraclass correlation coefficients with 95% confidence intervals, standard error of measurement, and minimal detectable change are presented in text at each stimulator output.

The reliability of both ΔAC and ΔTC can be found in Figures 4, 5, respectively. No significant effects of block on both ΔAC and ΔTC were detected within or between days (all P > 0.052). Both respiratory belts displayed good-to-excellent within-day reliability (ΔAC ICC range 0.75–0.94; ΔTC ICC range 0.77–0.98) at all stimulator outputs excluding 30% (both ICCs = 0.74). Between days, ΔAC and ΔTC showed moderate reliability at 20% (both ICCs = 0.70) and 30% (ICC = 0.73 and 0.74, respectively) of maximal stimulator output. At all other stimulator outputs, between-day reliability was good to excellent for ΔAC (ICC range 0.77–0.90) and excellent for ΔTC (ICC range 0.91–0.96).

### 3.2 Side-effects to rBAMPS

**FIGURE 6:**
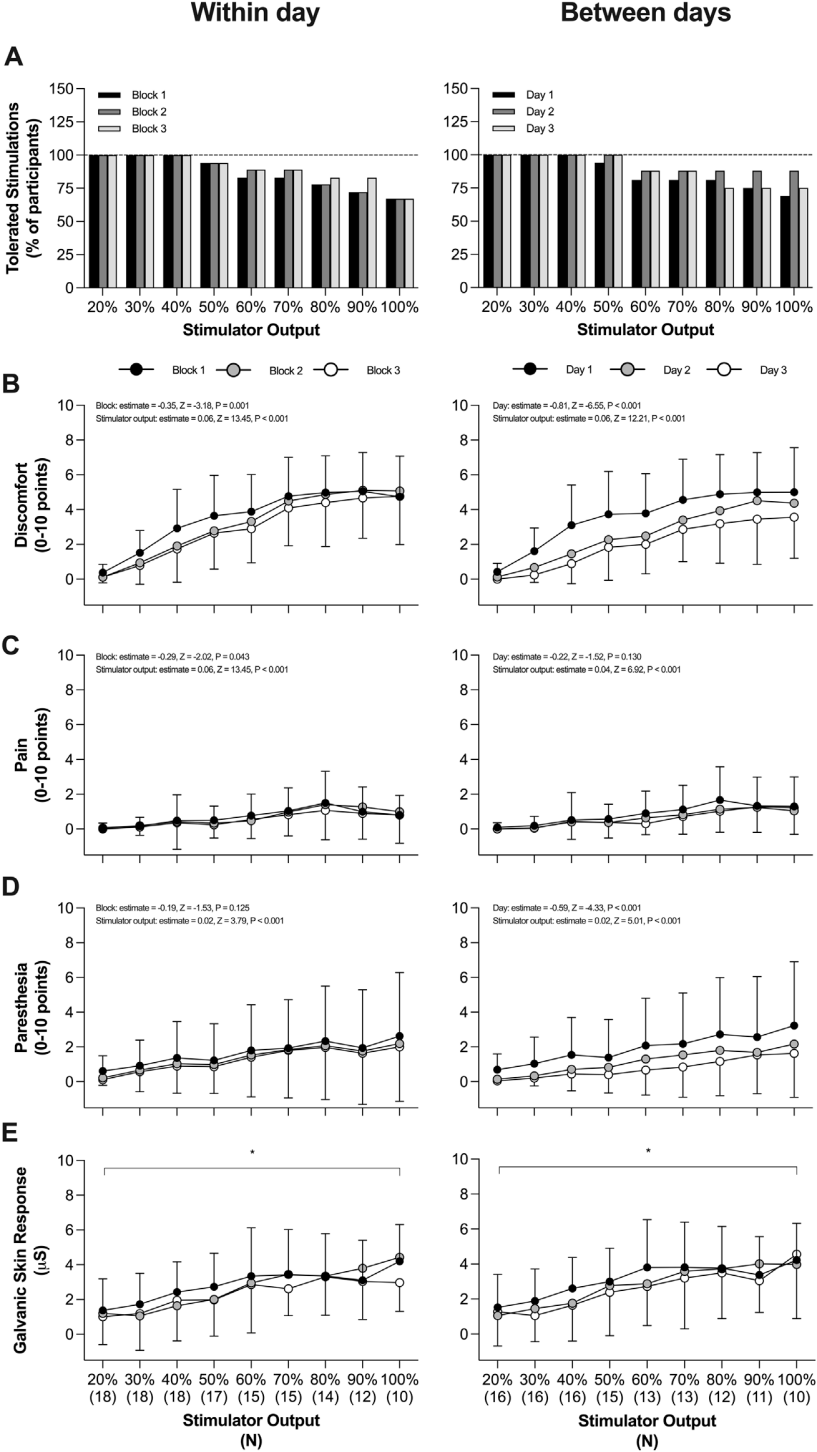
Side-effects in response to rapid bilateral anterolateral magnetic phrenic nerve stimulation. **(A)** Tolerability with increasing stimulator output (defined as participant undergoing three trains). **(B)** Discomfort within and between days. **(C)** Pain within and between days. **(D)** Paresthesia within and between days.**(B–D)** include plotted group data as mean and standard deviation with model coefficients from an ordinal regression presented in text. **(E)** Change in galvanic skin response within and between days. Plotted data represent group mean and standard deviation. Symbols on the panel correspond to the results of a mixed-effects model analysis such that ^*^, main effect of stimulator output. All panels P < 0.05.

All side-effects in response to rBAMPS including discomfort, pain, paresthesia, and ΔGSR are found in Figure 6. The within-day grand means for discomfort were 3.4 ± 1.5 (block 1), 3.0 ± 1.4 (block 2), and 2.6 ± 1.4 (block 3) points. Each subsequent rBAMPS block within day was associated with reduced discomfort (estimate = −0.35, Z = −3.18, P = 0.001), as well as less instances of participants selecting greater than 3 points (estimate = −0.33, Z = 2.12, P = 0.034, odds ratio [95% confidence interval] = 0.716 [0.526–0.975]). Grand mean discomfort values across days corresponded to 3.5 ± 1.6 on day 1, 2.3 ± 1.4 on day 2, and 1.8 ± 1.1 on day 3. Each subsequent day was also associated with reduced discomfort (estimate = −0.81, Z = −6.55, P < 0.001) and a reduced likelihood of participants selecting greater than 3 points (estimate = −0.79, Z = −4.67, P < 0.001, odds ratio = 0.453 [0.32–0.631]). Each subsequent rBAMPS block within day was associated with reduced pain (estimate = −0.29, Z = −2.02, P = 0.043), while subsequent days were associated with reduced paresthesia (estimate = −0.59, Z = −4.33, P = <0.001), as well as a reduced likelihood of rating paresthesia greater than three points (estimate = −0.66, Z = −3.19, P < 0.001, odds ratio = 0.518 [0.346–0.776]). Increasing discomfort, pain, and paresthesia (as well as increased instances of selecting greater than 3 points) were all significantly associated with increasing stimulator output both within and between days (all P ≤ 0.001).

No significant effect of block was detected on ΔGSR within- (P = 0.513) or between-days (P = 0.441). A significant effect of stimulator output was detected on ΔGSR both within- (P < 0.0001) and between-days (P = 0.002). The odds of experiencing at least one UAC within a day did not change with subsequent blocks (estimate = −0.14, Z = −1.06, P = 0.287, odds ratio = 0.867 [0.668–1.13]). However, the odds of experiencing at least one UAC decreased with subsequent days (estimate = −0.32, Z = −2.17, P = 0.030, odds ratio = 0.725 [0.542–0.969]). Within participants that experienced at least one UAC and were tested on all 3 days (13 of 16 participants), the mean percentage of UAC occurrences (expressed as percentage of total number of rBAMPS trains conducted on the participant) was on average 33% ± 33% on day 1, 22% ± 28% on day 2, and 22% ± 30% on day 3.

## 4 Discussion

The primary aim of the present study was to determine the reliability of rBAMPS-elicited inspiratory responses within and between days. In addition, we sought to determine if participants report reduced perception of discomfort, pain, and paresthesia during repeated sessions of rBAMPS. Our findings suggest that both P_di_ and V_T_ in response to rBAMPS are reliable at various stimulator outputs across sessions within and between days. It should be noted, however, that both relative and absolute indexes of reliability indicate a reduced reliability of rBAMPS between days compared to within days, especially at stimulator outputs exceeding 50% of maximum. Finally, our findings suggest that the application of rBAMPS became less painful and uncomfortable following repeated sessions within a day and became less uncomfortable and produced lesser sensations of paresthesia following repeated sessions across days.

### 4.1 Reliability

In order for rapid phrenic nerve stimulation to become part of the standard of care in the ICU, stimulations must produce effective diaphragmatic contractions, and these contractions need to be reliable over time. To our knowledge, no study has systematically assessed the reliability of P_di_ in response to rapid magnetic stimulation within and between days, while the reliability of P_di,tw_ elicited from a single stimulus has been explored at length (Criner et al., 1999; Luo et al., 2002; Ramsook et al., 2021). Ramsook et al. (2021) showed good relative within-day reliability of P_di,tw_ during cervical magnetic stimulation with an ICC value of 0.89, which is in line with our findings obtained through a variety of tested stimulator outputs (ICC range 0.78–0.89). The authors also showed good between-day reliability of P_di,tw_ with an ICC of 0.87, which is similar to the between-day reliability of P_di,mean_ responses in the present study between 20% and 50% of maximal stimulator output, but greater than responses at outputs from 60% to 100%. It is possible that the decreased reliability between days in the present study is in part due to the increased difficulty in placing the magnetic coils on subsequent days in the exact same position during bilateral anterolateral magnetic phrenic nerve stimulation compared to cervical magnetic stimulation. For instance, during bilateral anterolateral magnetic phrenic nerve stimulation, two coils need to get placed in the exact same position, while during cervical magnetic stimulation, a singular circular coil needs re-placement. With respect to bilateral stimulation, Criner et al. (1999) reported a within-day CV of 5.3% during bilateral electric phrenic nerve stimulation, while (Luo et al., 2002) reported a CV of 11% between days using bilateral anterolateral magnetic phrenic nerve stimulation. The present study reports greater variation during rBAMPS and is likely due in part to the additional movement that occurs during rapid stimulation compared to single twitches.

The present study tested the reliability of respiratory belts in response to rBAMPS due to their potential ability to be a non-invasive index of diaphragmatic contraction. Both ΔTC and ΔAC displayed good-to-excellent reliability at the majority of tested stimulator outputs both within and between days. Both respiratory belts displayed greater reliability compared to P_di_, but this may actually reflect that they are less responsive and sensitive in comparison to the balloon catheters. Indeed, previous work by our group showed a very poor correlation between ΔTC and P_di,mean_ (r = 0.11) and a moderate correlation between ΔAC and P_di,mean_ (r = 0.52) (Boyle et al., 2022), indicating neither belt has a strong relationship with the gold-standard measurement of P_di_. Conducting the same analysis in the present study reveals a moderate correlation between P_di,mean_ with both ΔTC (r = 0.50, P < 0.001) and ΔAC (r = 0.52, P < 0.001). The increased correlation we report here compared to the aforementioned study is likely due to the greater sample size. Regardless, both belts still only show a moderate level of correlation with P_di_ and their usefulness in quantifying diaphragmatic contractions still needs to be further explored.

### 4.2 Factors influencing reliability

There are a number of factors that likely influence the reliability of rBAMPS. For example, whether or not participants had the presence of UAC between days dramatically influenced the reliability of our reported data. Within the participants of the present study, two had consistent UAC during day 1 (resulting in the absence of V_T_ and large P_di_) and less instances of UAC during days 2 and 3 (resulting in the presence of a V_T_ and reduced P_di_ during some rBAMPS). Alternatively, one participant had many instances of UAC on days 2 and 3, but not day 1. By removing these select participants from analysis, all variables show increased test–retest reliability at all stimulator outputs, and the significant effect of day on P_di,mean_ is eliminated. For example, the lowest reported ICCs are P_di,mean_ between days at 70% (ICC = 0.57) and 90% (ICC = 0.56) of maximal stimulator output, which both increase to 0.89 and 0.84, respectively, when the participants with inconsistent UAC are removed from the sample. Finally, it should be noted that two additional participants had UAC during nearly every rBAMPS on all visits, and these participants do not alter the reliability results. As such, it is paramount to relieve UAC during rBAMPS to optimize the reliability of the technique in participants with inconsistent UAC responses. In a clinical context, the occurrence of UAC is likely irrelevant in intubated patients, while it still might be a concern when using rBAMPS during non-invasive mechanical ventilation.

Another factor that likely influences rBAMPS reliability is the placement and replacement of the magnetic coils. Within days, coils were only repositioned in a small subset of participants (N = 6) due to a bathroom break. When performing a sub analysis in those participants, ICCs did not change between the two blocks in which coils needed to be repositioned, compared to the two blocks where the coils remained stationary during the break. This may be due to the fact that coils could be easily repositioned within days as the coil position was marked with ink. Between days it is feasible to suggest that despite efforts of the authors to reposition the coils in the exact same position, it was not always the case, and in part reflects the decrease in P_di,mean_ reliability between days compared to within days. As such, finding the optimal coil position to stimulate the phrenic nerves each day is recommended in order to achieve similar responses.

### 4.3 Side-effects

The present study reports a reduction in discomfort and pain across rBAMPS blocks within day, as well as a reduction in discomfort and paresthesia between days. Previous research has reported habituation effects over repeated bouts of exposure to hot and cold stimuli (LeBlanc and Potvin, 1966; Bingel et al., 2007; Rennefeld et al., 2010) due to protective responses of the central nervous system. Despite this, we cautiously interpret our results and suggest that more consistent exposure to rBAMPS would need to be further explored to determine if a true habituation effect occurs. If a habituation to rBAMPS does exist, it would potentially allow patients to be stimulated at higher levels of stimulator outputs for a given level of pain or discomfort, or potentially increase their highest tolerated stimulator output over time. This would ultimately lead to increased stimulus to the diaphragm, potentially increasing protective effects.

### 4.4 Limitations

There are various limitations to consider in the present study. First, the participants undergoing stimulation were conscious healthy individuals. Whether responses are equally reliable in sedated patients needs to be established. In addition, how patients’ progress clinically may further influence the reliability of rBAMPS could not be accounted for in the present study. For example, a decrease in lung compliance or an increase in subcutaneous edema may both affect responses to rBAMPS. Second, the participants in the present study were not undergoing mechanical ventilation, and the interaction between rBAMPS and mechanical ventilation needs to be further explored in the context of reliability. Third, the present study performed 1-s rBAMPS at 25 Hz; thus, the generalizability of these results is not yet known. Finally, the present study only compared a total of nine rBAMPS trains at each level of stimulator output within and between days, which does not represent the likely application of rBAMPS in a clinical setting.

### 4.5 Conclusions

The present study reports rBAMPS as a tool to produce reliable diaphragmatic contractions. Despite this, to ensure patient safety, systems that automatically adjust stimulator output to avoid overinflation of the lung still need to be established. In addition, given the reliability of diaphragmatic contraction decreases between days compared to within days, stimulation parameters may need to be adjusted in order to achieve the same desired responses between days. Finally, rBAMPS appears to be a relatively painless experience that becomes more comfortable after repeated bouts.

## Data Availability

The raw data supporting the conclusions of this article will be made available by the authors, without undue reservation.

## Supplementary material

SUPPLEMENTAL FIGURE S1 Peak transdiaphragmatic pressure generation (P_di,peak_) in response to rapid bilateral anterolateral magnetic phrenic nerve stimulation. Single-participant data points are represented by white circles connected by dotted lines. Upper and lower bands of the underlying box plots represent the interquartile ranges, while the black line represents the median. Box plot whiskers represent min and max values. Number of participants, intraclass correlation coefficients with 95% confidence intervals, standard error of measurement, and minimal detectable change are presented in text at each stimulator output.
